# Exotic Vegetable Oils for Cosmetic O/W Nanoemulsions: *In Vivo* Evaluation

**DOI:** 10.3390/molecules21030248

**Published:** 2016-02-24

**Authors:** Tatiana A. Pereira, Carolina M. Guerreiro, Monica Maruno, Marcio Ferrari, Pedro Alves Rocha-Filho

**Affiliations:** 1 Department of Pharmaceutical Sciences, Faculty of Pharmaceutical Sciences of Ribeirão Preto, University of São Paulo, Avenida do Café, s/n, Bairro Monte Alegre, Ribeirão Preto, SP 14040-903, Brazil; tatianapereira1@gmail.com (T.A.P.); guerreiro.carolina@yahoo.com.br (C.M.G.); 2 Pharmacy Course Coordination, Centro Universitário Barão de Mauá, R. Ramos de Azevedo, 423, Jardim Paulista, Ribeirão Preto, SP 14090-180, Brazil; monica.maruno@baraodemaua.br; 3 College of Pharmacy, Federal University of Rio Grande do Norte, Rua Gustavo Cordeiro de Farias, s/n, Petrópolis, Natal 59012-570, RN, Brazil; ferrarimarcio@uol.com.br

**Keywords:** nanoemulsions, raspberry seed oil, passion fruit oil, peach oil, lanolin, skin hydration

## Abstract

Oil-in-water nanoemulsions are stable systems with droplet sizes in the 20–200 nm range. The physicochemical properties of these systems may be influenced by the addition of additives. Thus, the influence of ethoxylated (EL) and acetylated lanolin (AL) addition on the droplet size, pH values, electrical conductivity and stability of nanoemulsions was investigated. Then, effect of nano-emulsions additives with EL (NE-EL) or AL (NE-AL) in hydration, oiliness and pH of the skin were evaluated. Nanoemulsion safety was evaluated through the observation of no undesirable effects after skin formulation application. Both additives caused changes in droplet size and electrical conductivity, but not in pH values. Nanoemulsions containing up to 6.0% ethoxylated lanolin and 2.0% acetylated lanolin remained stable after centrifugation tests. Higher concentrations of the additives made the nanoemulsions unstable. Stability tests showed that ethoxylated lanolin produced more stable nanoemulsions then acetylated lanolin and that the major instability phenomenon occurring in these systems is coalescence at elevated temperatures. Nanoemulsion-based lanolin derivatives increased skin hydration and oiliness and did not change cutaneous pH values. These formulations are non-toxic since they did not cause any irritation on the skin surface after nanoemulsion application, showing potential as carriers for pharmaceuticals and cosmetic applications.

## 1. Introduction

Recently there has been an increased interest of the pharmaceutical and cosmetic industries in nanoemulsions due to some characteristics such as high kinetic stability, actives release efficiency on skin, excellent sensorial and esthetic aspects besides the need for less surfactant (5%–10%) thus reducing the possibility of skin irritation and production costs, when compared to microemulsions [1,2]. Nanoemulsions are a class of emulsions that have droplet sizes in the 20–200 nm range and are therefore transparent or translucent. Some researchers [1,2] have studied the stability of 15 year old nanoemulsions and concluded that they remained stable over that period, not showing alterations in droplet size. Some studies show that surfactant blends are more effective than single ones. The emulsification process can affect the droplet size, its distribution and therefore the stability of nano-emulsions [3,4,5,6,7,8]. In addition, the composition can influence the physical characteristics and consequent stability of nanoemulsions [9]. Lanolin (from the Latin *lana*, wool) is a complex substance formed by a mixture of esters from numerous combinations of long chain alcohols (C7 to C40) and fatty acids (C14 to C36) derived from the sebaceous gland of sheep and accumulated in these animals’ wool. In the pharmaceutical industry, lanolin is employed as a vehicle for drugs and cosmetics due to its favorable properties like low toxicity, occlusive capacity and maintenance of skin hydration by water retention [3]. However, there are no reports in the literature about the influence of the use of lanolin derivatives on the physicochemical characteristics and stability of nanocarriers as well as in its moisturizing activity. The aim of this work was to evaluate the influence of lanolin derivatives (ethoxylated and acetylated lanolin) on the physicochemical characteristics and stability of vegetable oil-based nanoemulsions.

## 2. Results and Discussion

### 2.1. Preparation of Nanoemulsions

The required HLB value of the mixture of oils (raspberry: passion fruit: peach oils, 1:1:1) was previously determined and found to be 9.0 [10]. Several pairs of surfactants (Table 1) were tested aiming to produce stable nanoemulsions with the smallest droplets. All proposed surfactant pairs produced characteristic translucent and bluish nanoemulsions with mean droplet diameter sizes smaller than 200 nm (Table 2). All nanoemulsions remained stable after centrifugation tests.

The thermal stress test results are presented in Table 3. We can observe that all nanoemulsion formulations were stable at 50 °C and changed little until 80 °C, while that numbered 4 remained stable until 80 °C.

The crucial step in the formation of nanoemulsions is the transition from W/O to O/W. It occurs through the formation of a bicontinuous or lamellar structure that depends on the system’s interfacial tension and on the amount of surfactants which has to be able to solubilize the oily phase at the phase inversion point [11,12].

An inadequate amount of surfactants can produce larger droplets with heterogeneous distribution [13,14]. The amount of surfactants employed (10.0%) was enough to produce nanoemulsions with small droplets, being the smallest droplet size that of nanoemulsions produced with the sorbitan monooleate/PEG 36 castor oil pair with a mean size of 48.0 ± 7.2 nm and monomodal distribution. Droplets with mean size of 54 nm using sorbitan monooleate and polysorbate 80 (8.00%) as non-ionic surfactant pair and the phase inversion production method, were reported by Liu *et al.* [15]. According to the previous reports [7,11,15], nanoemulsions with mean droplet sizes below 200 nm are formed if the oil/surfactant ratio and the required HLB value are optimized. The final droplet diameter size is determined by the characteristic distance of the intermediary bicontinuous or lamellar phase formed at the inversion point and that distance depends on the employed oil/surfactant and surfactant/surfactant ratios [12].

### 2.2. Physicochemical Characterization

#### 2.2.1. Granulometry (Diameter Mean Size)

**Figure 1:**
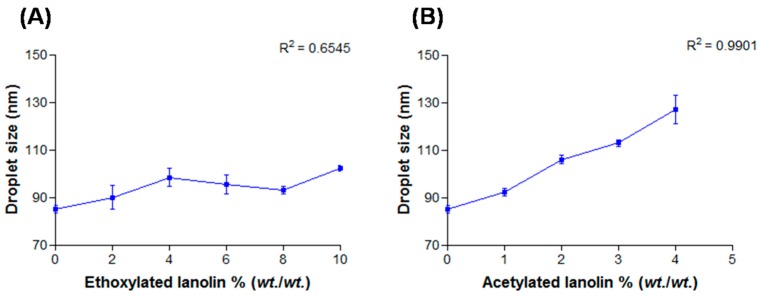
Droplets mean size as a function of lanolin derivatives concentration: (**A**) EL and (**B**) AL.

On emulsions added with AL the relation between droplet size and additive concentration is linear (Figure 1B, R^2^ = 0.9901) but not on those added with EL (Figure 1A). The pronounced increase observed in emulsions with AL may be due to the increase of the oil/surfactant ratio also reported by Maestro *et al.* [14]. EL has ethylene oxide molecules and therefore hydrophilic character which may explain the maintenance of oil/surfactant ratios in systems containing it.

#### 2.2.2. Interfacial Tension

The droplet size is linked to the interfacial tension of the system, so that the surfactant concentration leads to its decrease and consequently to that of droplets [4]. According to the interfacial tension theory, surfactants are adsorbed on the liquid interface allowing the system emulsification. Thus, interfacial tension measurements are frequently used to observe surfactants adsorption on emulsions interface [16,17].

**Figure 2:**
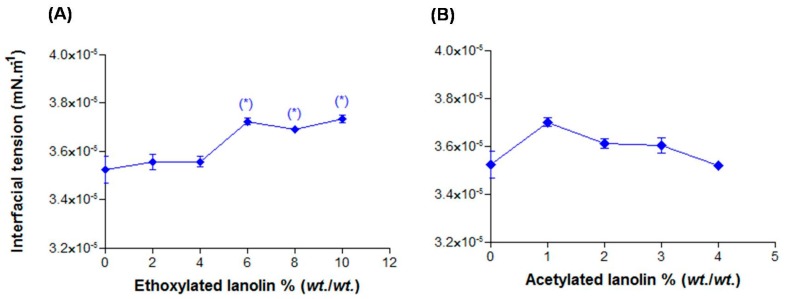
Interfacial tension in function of the lanolin derivatives: (**A**) EL and (**B**) AL. * *p* > 0.05.

A small increase on the interfacial tension could be observed on systems added with EL (Figure 2A) which was statistically significant after 6.0%. As an amphiphilic substance, EL is organized on the O/W interface and may interfere with the surfactant adsorption which may have caused the interfacial tension increase as well as the droplet size change. AL did not significantly modify (*p* > 0.05) the interfacial tension (Figure 2B), probably because AL is a lipophilic substance and therefore is organized inside the droplet not interfering with interfacial tension.

#### 2.2.3. Electrical Conductivity

**Figure 3:**
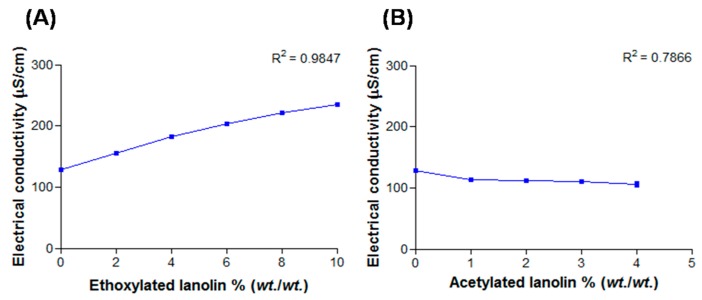
Electrical conductivity values in function of lanolin derivatives: (**A**) EL and (**B**) AL.

Electrical conductivity is a parameter used to identify the type of emulsion, determining phase inversion temperature as well as identification of possible instability phenomena such as flocculation and coalescence. The conductivity of nanoemulsions added with EL was dependent on the concentration (Figure 3) (R^2^ > 0.9). The increase in conductivity can be due to increased concentration of lanolin, a water soluble substance with a tendency to conduct electrical current. In nanoemulsions added with AL (Figure 3B) there was no linear relationship between conductivity and concentration of acetylated lanolin. This was expected, since AL addition results in increased oily fraction instead of aqueous, responsible for conducting electrical current. Although the addition of lanolin derivatives caused changes in electrical conductivity, these changes did not result in instability of the nano-emulsions.

#### 2.2.4. pH Values

The addition of lanolin derivatives did not cause changes in pH values of the nanoemulsions (*p* > 0.05) in both EL and AL added systems, suggesting that the different chemical groups in the additives did not interfere with the initial nanoemulsion pH values.

### 2.3. Lanolin Derivatives’ Addition

**Figure 4:**
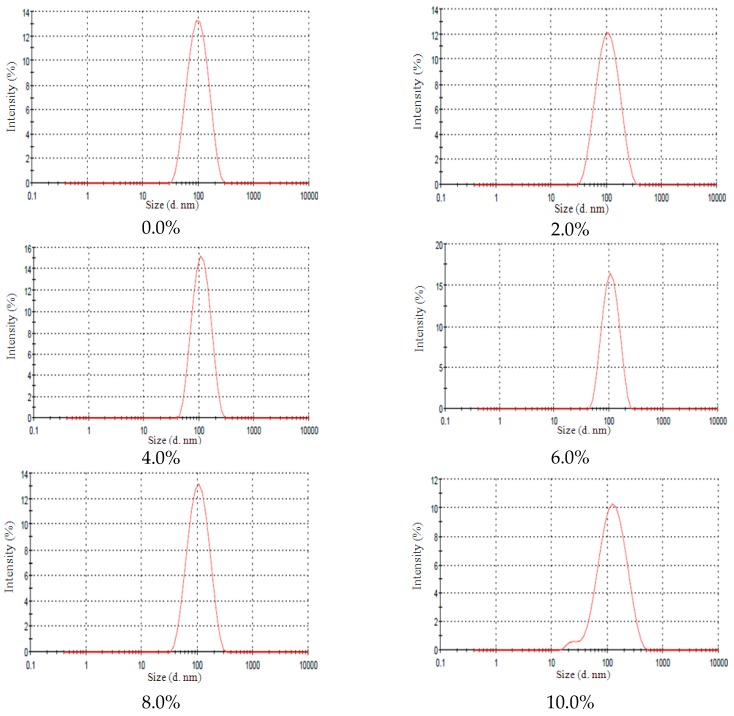
NE-EL size distribution at different EL concentrations (0.0%; 2.0%; 4.0%; 6.0%; 8.0% and 10.0%).

The nanoemulsions remained macroscopically stable with lanoline derivatives and no instability signs were observed after 24 h. However, when submitted to centrifugation, the nanoemulsions with 8.0% and 10.0% of EL presented instability signs after the third cycle which can be observed by a broad particle size distribution curve at 10.0% EL concentration. The size distribution of NE-EL at different concentrations can be observed in Figure 4. Yang *et al.* [18] verified that the addition of alcohol enhances the continuous phase density and the authors observed a variation in droplet sizes but no evident instability signs of the nanoemulsions. The addition of an ethoxylated alcohol lanolin derivative at elevated concentrations may increase the continuous phase’s density which can speed up the flocculation process and therefore accelerate the phase separation, so this behavior seems to be dependent of the additive concentration since only at high amounts (8.0 and 10.0%) was nanoemulsion instability observed.

**Figure 5:**
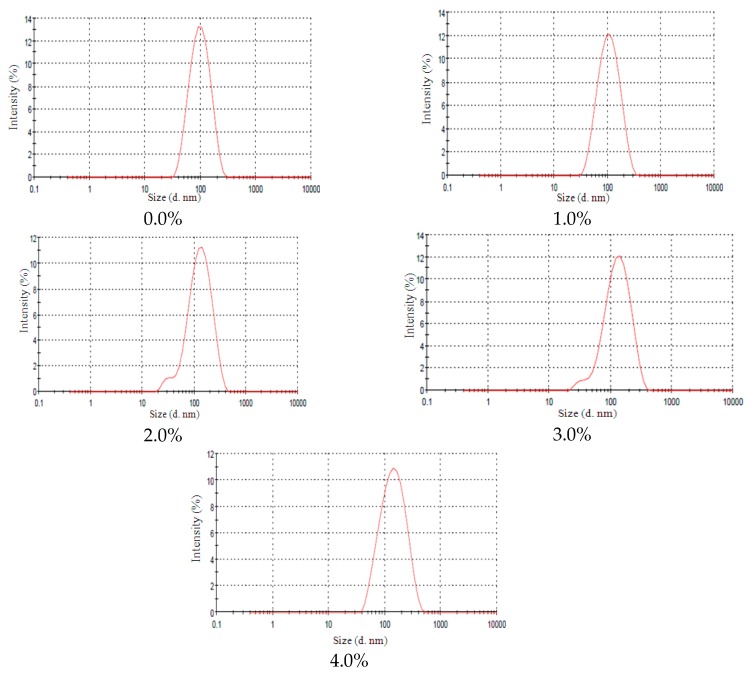
NE-AL size distribution at different EL concentrations (0.0%; 1.0%; 2.0%; 3.0%; 4.0% and 5.0%).

In systems added with AL, the formulation containing 5.0% of this additive presented phase separation 24 h after preparation and those with 3.0% and 4.0% showed creaming after the third centrifugation cycle. One of the main factors for the nanoemulsion system occurrence is the critical amount of surfactant needed to coat newly formed droplets during the emulsification process, inhibiting the coalescence. Thus, there should be a suitable oil/surfactant ratio for the formation of droplets [12,19]. The change in this critical ratio leads to an increase in droplet size and a consequent broad droplet size distribution curve, as observed in Figure 5. Therefore, AL being a lipophilic substance can lead a modification in the oil/surfactant ratio, resulting in a lower amount of available surfactant to solubilize the oil [12]. This effect becomes more pronounced with increasing concentration of AL, leading to total separation of phases at higher concentrations of the additive (5.0%). Moreover, AL has a function of solubilizer and may lead to a decreased amount of available surfactant to adsorb at the interface O/W of the nanoemulsion, resulting in decreased stability of such systems with high concentrations of AL. In both cases, we observed that this effect is dependent on the concentration of lanolin derivatives. At low concentrations, it is possible to form stable nanoemulsions and as the concentration increases, reversible instability phenomena appear until phase separation occurs at higher concentrations (5.0%).

### 2.4. Stability Tests

Parameters such as droplet size, pH values and conductivity as a function of time are able to predict the stability of a nanoemulsion in a given period of time. The addition of lanolin derivatives to nanoemulsions can cause changes in their physicochemical characteristics as globule size, surface tension and electrical conductivity but these changes do not result in intrinsic instability of the systems. In light of these results, nanoemulsions composed of raspberry, passion fruit and peach oils (1:1:1), sorbitan monooleate/PEG 36 castor oil, 2.0% AL or 6.0% EL were submitted to accelerated stability tests. In both cases, the selected formulations were those which had the highest amount of lanolin derivatives (AL and EL) and remained stable because at higher emollient amount, moisturizing effects are more pronounceable.

**Figure 6:**
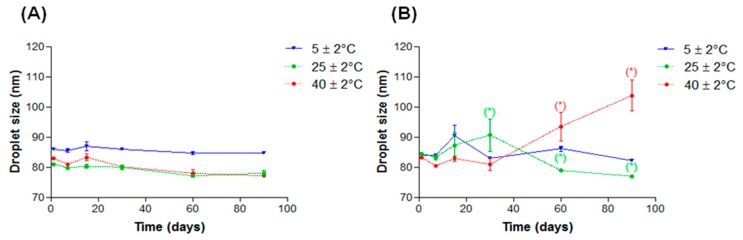
Droplet mean sizes in function of time of nanoemulsions with added lanolin derivatives subjected to different storage temperatures: (**A**) EL and (**B**) AL. * *p* > 0.05.

**Figure 7:**
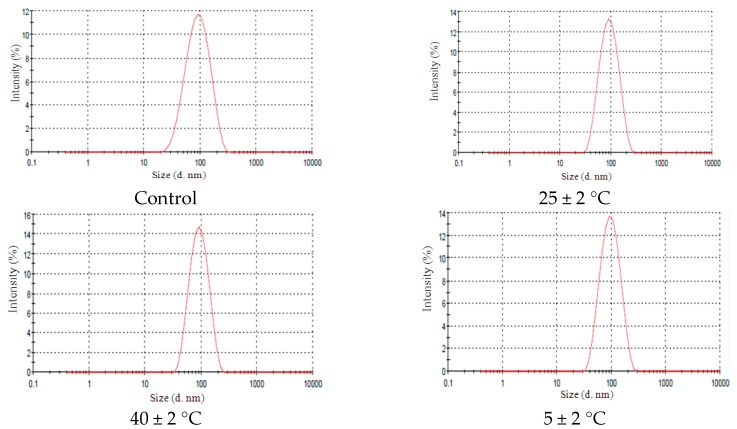
Narrow droplet size NE-EL 6% distribution under different conditions after 90 days of storage. Control curve refers to initial time (0 day), 25 ± 2 °C refers NE-EL 6% stored at room temperature. 40 ± 2 °C refers NE-EL 6% stored at high temperatures and 5 ± 2 °C refers NE-EL 6% stored at low temperatures.

To be considered suitable for pharmaceutical/cosmetic use, an emulsion needs to be classified according to some criteria such as prolonged stability under various conditions of storage [2]. For the systems added with 6.0% of EL no statistical significant change was observed in droplet size (*p* > 0.05) after 90 days under the different storage conditions (Figure 6A), which showed narrow droplet distribution (Figure 7). This stability may be attributed to the ethylene oxide groups present in this additive which could cause a steric hindrance avoiding mechanical shock between droplets and inhibiting coalescence [20]. In addition, EL is a highly viscous substance. The influence of addition of tocopherol, another highly viscous substance, in nano-emulsions was studied by Teo *et al.* [21]. And the authors concluded that this additive improves the stability due to the high viscosity.

**Figure 8:**
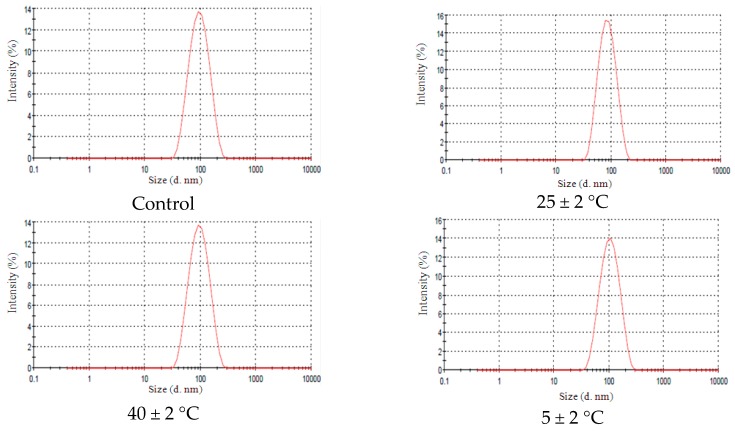
Narrow droplet size NE-AL 2% distribution under different conditions after 90 days of storage. Control curve refers to initial time (0 day), 25 ± 2 °C refers NE-EL 6% stored at room temperature. 40 ± 2 °C refers NE-EL 6% stored at high temperatures and 5 ± 2 °C refers NE-EL 6% stored at low temperatures.

In systems added with 2.0% of AL increase in the size droplet there was a statistically significant (*p* < 0.05) when those formulations were stored after 60 days at 40 ± 2 °C (Figure 6B). But, no change in narrow droplet size distribution curve was observed (Figure 8).

**Figure 9:**
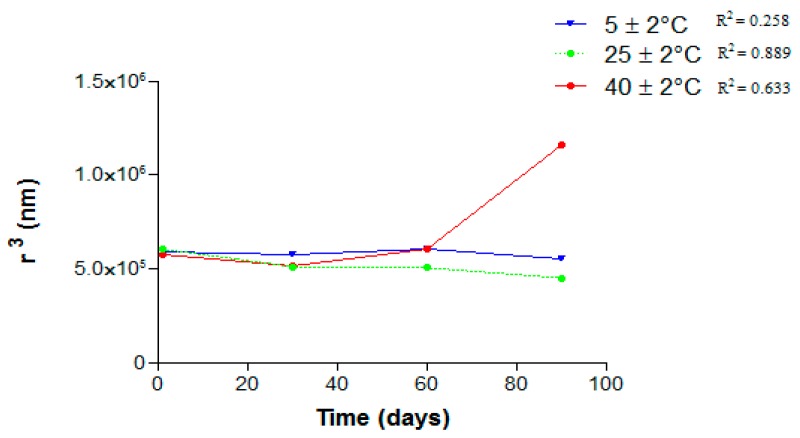
r^3^ in function of time of NE-AL 20%.

No linear relationship was observed between r^3^ and time, indicating that the instability phenomena that lead to the droplet size increasing is coalescence (Figure 9). High temperatures can decrease the systems’ apparent viscosity and increase the kinetic motility of the dispersed phase or surfactant agent. This could promote creaming and droplets coalescence [22]. When those nanoemulsions were stored at 25 ± 2 °C there was an increase of the droplet sizes followed by a decrease over time. Surfactants present in the system may be adsorbed on the droplet surface or dispersed in the aqueous solution as micelles or monomers. Molecules of the oil can be transferred from the inner phase to outer aqueous phase, causing droplets shrinking. Another phenomenon that may be occurring is a transfer of mass from small droplets to larger ones, called Ostwald ripening. The changes in the droplets sizes are a balance between solubilization and mass transfer, so as a rate overcomes the other, we can observe a decrease or increase of the droplet size [23].

**Figure 10:**
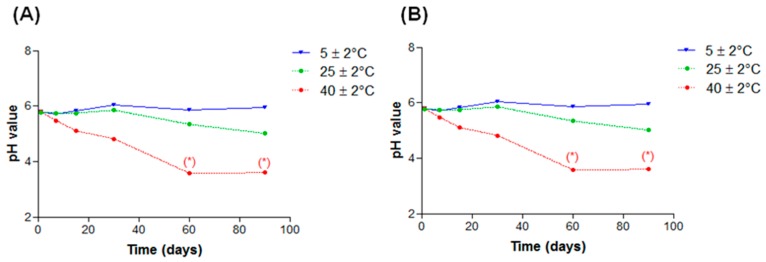
pH values in function of time of nanoemulsions subjected to different storage temperatures: (**A**) EL and (**B**) AL. * *p* < 0.05.

In nanoemulsions added with both lanolin derivatives, the pH values did not showed changes when the formulations were stored at 5 ± 2 °C or 25 ± 2 °C. However, those stored at 40 ± 2 °C showed a statistically significant (*p* < 0.05) decrease in the pH values after 60 days (Figure 10A,B). According to Masmoudi *et al.* [24] a drop in pH values may represent an oily phase oxidation with hydroperoxide formation or triglyceride hydrolysis leading to fatty acid formation and therefore a drop in pH values. Oxidative reactions are responsible for alterations that compromise the integrity and safety of products making them inappropriate for use. Light incidence and high temperatures can accelerate this process [25]. Although nanoemulsions presented changes only when submitted to high temperatures, an antioxidant could be added to the formulation to guarantee stability for a longer period of time.

**Figure 11:**
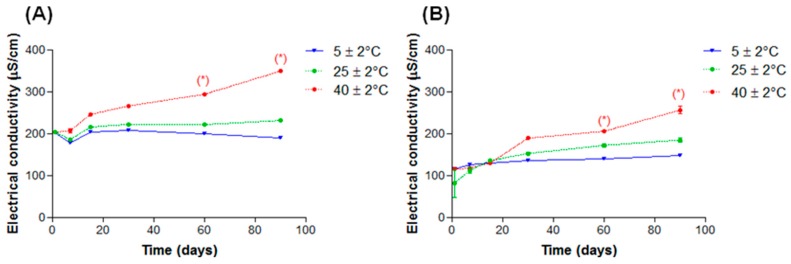
Electrical conductivity in function of time of nanoemulsions subjected to different storage temperatures, (**A**) EL and (**B**) AL. * *p* < 0.05.

A variation in electrical conductivity of nanoemulsions may be related to instability processes. We could observe an increase in conductivity values after 60 days (*p* < 0.05) when samples were kept at 40 ± 2 °C in nanoemulsions containing both lanolin derivatives (Figure 11A,B).

Electrical conductivity and pH values alterations occurred in both systems. In systems added with EL it was probably due to oxidation and hydrolysis reactions considering that no droplet size variation was observed. In systems added with AL the variation in electrical conductivity may also be due to droplet coalescence.

According to Masmoudi *et al.* [24] it’s difficult to evaluate formulations’ stability only by electrical conductivity because there is no linear correlation between this parameter’s increase values and instability phenomena. Thus, although the electrical conductivity alterations that occurred under high temperature (40 ± 2 °C), these nanoemulsions can be considered stable.

Through stability tests we found that EL plays an important role on emulsions’ stability and that low temperatures (5 ± 2 °C) contribute to that. These results confirm those of Ee *et al.* [26] that affirm that there is an optimal storage temperature where emulsions maintain stability for a longer time. The formulations studied here can be stored at 25 ± 2 °C because they showed minimal alteration under this condition.

### 2.5. In Vivo Nano-Emulsions Evaluation: Hydration, Oiliness and pH Skin Evaluation

In the cosmetic area, skin hydration and anti-aging effect affect directly the development of new products and they are, therefore, important criteria in this process. The moisturizing capacity of a cosmetic product can be influenced by excipients or active ingredients that are incorporated into the formulation [27].

**Figure 12:**
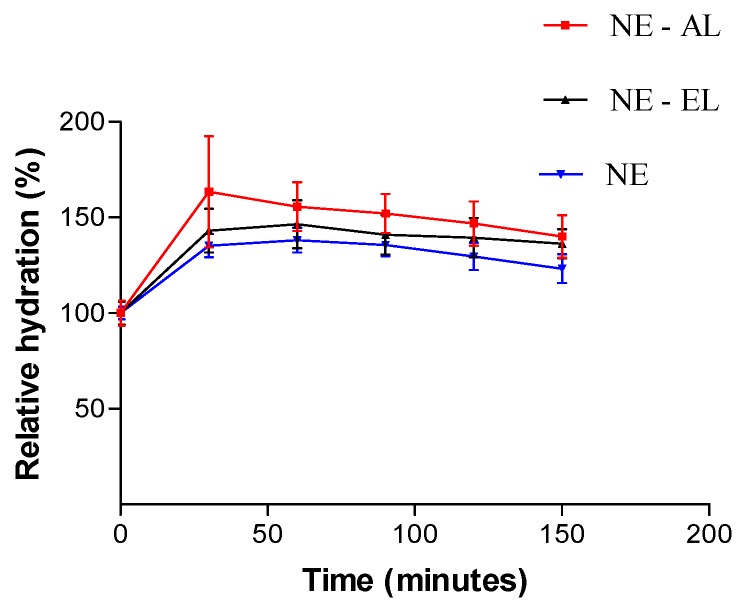
Relative hydration of the *stratum corneum* in function of time after nanoemulsion application.

Both NE-AL and NE-EL showed a tendency to enhance *stratum corneum* hydration related to NE which had no lanolin derivatives (Figure 11). Although in Figure 12 it is possible to observe that NE-AL promoted greater hydration then NE-EL, there is no significant differences between them (*p* > 0.05). The results are in accordance with our expectance, since lanolin has emollient properties with high water absorption capacity, showing greater relative hydration related to nanoemulsions without lanolin derivatives.

**Figure 13:**
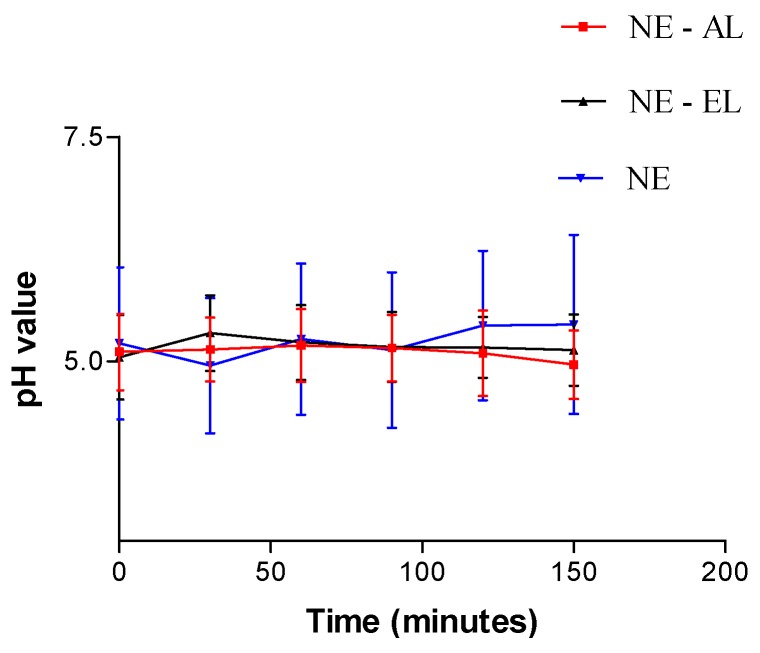
Skin pH values in function of time after nanoemulsion application.

No significant differences were observed in skin pH values, which remained around 5.0 after skin nanoemulsion application (*p* > 0.05; Figure 13). It indicates that the formulations are suitable for cosmetic use since they do not compromise the barrier function of the skin.

**Figure 14:**
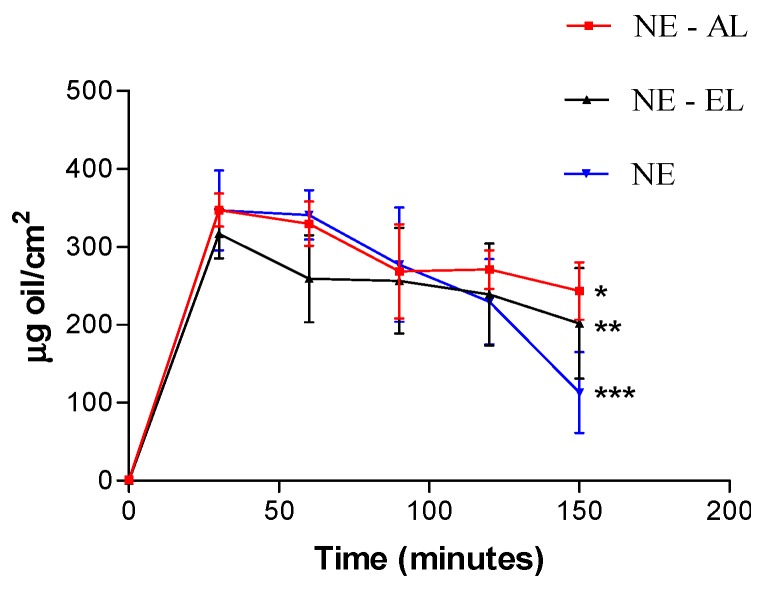
Skin oiliness in function of time after nanoemulsion application. * *p* < 0.05 vs NE-AL in time 0, 30, 60, 90 and 120 min. ** *p* < 0.05 *vs.* NE-EL in time 0, 30, 60, 90 and 120 min. *** *p* < 0.05 *vs.* NE in time 0, 30, 60, 90 and 120 min.

All tested formulations increased skin oiliness significantly (*p* < 0.05) at all tested times (Figure 14). After 120 min formulations without lanolin derivatives presented a decrease in the oiliness values showing that lanolin derivatives (AL and EL) increase formulation skin adhesiveness. Thus, formulations containing lanolin derivatives remain for a longer time on the skin surface related to nanoemulsions without any lanolin derivatives. There were no statistically significant differences in the skin oiliness increase as a function of time among the formulations (*p* > 0.05).

**Figure 15:**
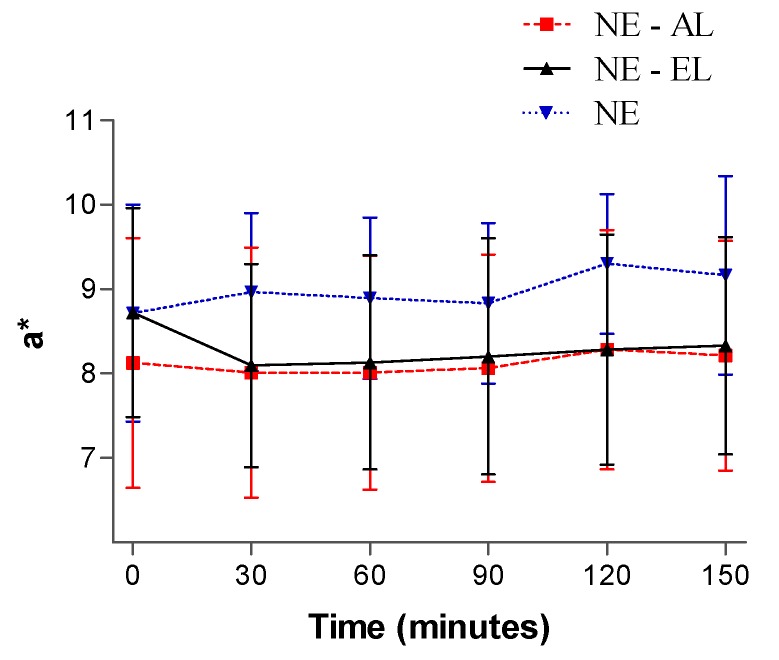
Colorimetric results in function of time after nanoemulsion application.

The results of color variation (a* parameter) did not reveal statistically significant alterations (*p* < 0.05) as a function of time for any of the tested nanoemulsions, indicating that they did not promote irritation of the skin during the tests (Figure 15).

## 3. Materials and Methods

### 3.1. Materials

Oils: *Rubus idaeus* (*Raspberry seed Oil); Passiflora edulis seed oil) Prunus persica* (*Peach kernel oil*) (INCI names and cosmetic grade) were obtained from Lipo chemicalsdo Brasil (São Bernando do Campo, SP, Brazil), Croda do Brasil (Campinas, SP, Brazil) and Pharmaspecial (Itapevi, SP, Brazil), respectively. Sorbitan monoloeate (Span^®^80-HLB = 4.3), PEGs 15 (HLB = 8.3), 30 (HLB = 11.7), 36 (HLB = 12.6), 40 (HLB = 13.0) and 54 (HLB = 14.4) castor oils were obtained from Oxiteno (São Paulo, SP, Brazil)). The lanolin derivatives Crodalan LA (acetylated lanolin) and Super Solan Pastilles (ethoxylated lanolin) were obtained from Croda do Brazil (Campinas, SP, Brazil). Freshly purified water was obtained from reverse osmosis equipment. All materials were employed as received without any additional purification process.

### 3.2. Methods

#### 3.2.1. Formulation of Nanoemulsions

A mixture of raspberry, passion fruit and peach oils (1:1:1) was employed as oily phase. Firstly blends of lipophilic (sorbitan monooleate) and hydrophilic castor oil derivatives: PEGs 30-, 36-, 40- and 54- castor oil were employed as surfactants. Secondly PEG-15 hydrophilic castor oil was associated to others PEGs 30-, 36-, 40- and 54- castor oil derivatives as surfactants.

##### Nano-Emulsions’ Obtention

The nano-emulsions was obtained by a low energy Emulsion Phase Inversion (EPI) method, so, aqueous and oily phase were both heated separately until 75 ± 5 °C and the aqueous phase was slowly spilled over the oily one containing the surfactant blend, under stirring (600 rpm) until the system reaches room temperature (25 ± 5 °C) [6].

##### Centrifugation Test

Five g of each nanoemulsion were submitted to centrifuge cycles (70 g; 440 g and 863 g) during 15 min each, at room temperature (25 ± 5 °C). Only formulations that did not show creaming or phase separation signs (n = normal or sl = slightly modified) were considerate stable. The tests were performed in triplicate.

##### Thermal Stress

The emulsions were subjected to thermal stress in a thermostated bath at a temperature range of 40 ± 2 °C to 80 ± 2 °C, increasing the temperature in 5 °C steps. The samples were held at each temperature for 30 min and then evaluated macroscopically. For this test, the following nomenclature was used to classify them: N = Normal; without change; LM = Slightly Modified; M = Modified; IM = Heavily Modified [28].

#### 3.2.2. Nanoemulsion Physicochemical Characterization

##### Diameter Mean Size

The droplets mean size and the polydispersity index were determined by photon correlation spectroscopy in a Malvern Zetasizer ZS (Malvern Instruments, Worcestershire, UK). The analyses were made in a 90° angle (triplicate) at room temperature (25 ± 5°C).

##### pH Values Determination

The pH values were determined at room temperature (25 ± 2 °C) by submerging the electrode (Digimed DM 20, Digimed, São Paulo, SP, Brazil) directly in the sample. The tests were performed in triplicate.

##### Electrical Conductivity Measurements

At room temperature (25 ± 2 °C) the electrode (mCA 150, Tecnopon, Piracicaba, SP, Brazil) was immersed directly in the sample. The tests were performed in triplicate. 

##### Interfacial Tension

This test was made according to the Du Nouy method and with a Fisher Scientific model 20 (Loughborough, Leicestershire, UK) tensiometer using a platinum rigid ring below room temperature (25 ± 2 °C).

#### 3.2.3. Lanolin Derivatives’ Addition in Nanoemulsions

To the stable nanoemulsion (NE) were added separately acetylated lanolin (AL) resulting in nanoemulsions containing acetylated lanolin (NE-AL) at 1.0%, 2.0%, 3.0%, 4.0% and 5.0% in the aqueous phase and ethoxylated lanolin (EL) resulting in nanoemulsions containing ethoxylated lanolin (NE-EL) at 2.0%, 4.0%, 6.0%, 8.0% and 10.0% in the oily phase. Then, the nanoemulsions were obtained by the abovementioned EPI method. These concentrations are the most commonly used of those substances for cosmetic systems. The composition of oil phase and surfactants ratio (sorbitan monooleate/PEG 36 castor oil) was kept constant.

##### Physicochemical Characterization after Lanolin Derivatives’ Addition

Diameter mean size, interfacial tension, pH values and electrical conductivity were evaluated as a function of the lanolin derivatives concentration. All tests were performed in triplicate.

##### Stability Tests

Nano-emulsions were subjected to different storage temperatures (25 ± 2 °C, 5 ± 2 °C and 40 ± 2 °C) during 90 days and their physicochemical characteristics (diameters mean size, pH values, electrical conductivity) were evaluated at regular time intervals of 7, 15, 30, 60 and 90 days or until instability was observed.

#### 3.2.4. *In Vivo* Nanoemulsion Evaluation: Hydration, Oiliness and pH skin Evaluation

After approval by the Ethics Committee on Human (Protocol n^r^ 146-FCFRP-USP, 25 August 2009), nanoemulsion dermal activity assessment was performed with controlled relative humidity (75% ± 5%) and temperature (24 ± 2 °C) in a room.

(a) Inclusion and exclusion criteria for selecting volunteers

Twenty healthy women aged between 21 and 35 years and free of skin care products were selected. Volunteers with skin diseases or hypersensitivity to any component of the formulation were not accepted.

(b) Application of formulations

The demarcated volunteers’ forearm areas were divided into groups according to the following treatments (in triplicate):

(1) nanoemulsions containing ethoxylated lanolin (6.0%);
(2) nanoemulsions containing acetilated lanolin (2.0%);
(3) nanoemulsions without additives.

Nanoemulsion (50 μL) was applied to the right forearm in three rectangular areas of 13.80 cm^2^ and distributed for 20 s by rubbing the test area using a circular motion [29]. Excess nanoemulsion was left on the skin and the assessment was made after 30, 60, 90 and 150 min of application. Hydration measures, pH and skin oiliness were performed in triplicate in each demarcated area with Corneometer^®^ CM 820 equipment (Courage & Khazaka Electronic GmbH, Köln, Germany).

##### Hydration Skin Evaluation

Hydration units cover 0–150 arbitrary units (AU), where 0 corresponds to very dry skin and 150 to the very skin hydrated. Skin hydration was calculated by the following equation:

(1) $$HR\%\;=\;\frac{100\;\times \;M_{p}\;}{M_{c}}$$

where: *HR*% = relative hydration; *M_p_* = average capacitance readings of product application areas; *M_c_* = average capacitance of the readings of the control region.

##### Skin pH Value Assessment

The study was conducted using pHmeter Skin^®^ PH 900 equipment (Courage + Khazaka).

##### Skin Oiliness Evaluation

The equipment used for the determination of skin oils was a Sebumeter^®^ ( Courage & Khazaka Electronic GmbH, Köln, Germany) and the result is expressed in g oil/ cm^2^.

##### 3.2.5. Irritant Potential Analysis

The study was conducted using Chromameter CR-200 equipment (Konica Minolta, Tokyo, Japan), using the Lab system color.

### 3.3. Statistical Analysis

The results were presented as mean ± standard deviation. Variance values analysis were analyzed using ANOVA followed by post hoc analysis using a Tukey test with *p* < 0.05 as the minimum level of significance using PRISM 5.0 software (Graph Pad, San Diego, CA, USA).

## 4. Conclusions

Lanolin derivative addition to previously obtained nanoemulsions caused alterations of droplet size and electrical conductivity of the systems, however, such changes do not compromise their stability, since the droplet size remains within the nanometer range (20–200 nm). Nanoemulsions added with EL were more stable than those with AL. Both lanolin derivatives increased skin hydration and oiliness, and did not change cutaneous pH valued. Nanoemulsions based on vegetable oils and lanolin derivatives are non-toxic since they did not cause any irritation on the skin surface after nanoemulsion application, being therefore, recommendable for use as vehicles for pharmaceuticals and cosmetics.

## Figures and Tables

**Table 1:** Surfactant blend concentrations (*w*/*w* %) to nanoemulsion preparation, maintaining constant the oil phase at 10.0 (*w*/*w* %) and purified water at 80.0 (*w*/*w* %).

Formulation	Sorbitan Monooleate	PEG-15 Castor Oil	PEG-30 Castor Oil	PEG-36 Castor Oil	PEG-40 Castor Oil	PEG-54 Castor Oil
1	3.65	-	6.35	-	-	-
2	4.34	-	-	5.66	-	-
3	4.60	-	-	-	5.40	-
4	5.34	-	-	-	-	4.66
5	-	7.93	2.07	-	-	-
6	-	8.36	-	1.64	-	-
7	-	8.51	-	-	1.49	-
8	-	8.83	-	-	-	1.15

**Table 2:** Droplet mean diameter size produced with different pairs of surfactants (HLB = 9).

Formulation	Mean Diameter Size (nm ± SD) *
(1) Sorbitan monooleate/PEG 30 Castor oil	51.0 ± 5.2
(2) Sorbitan monooleate/PEG 36 Castor oil	48.0 ± 7.2
(3) Sorbitan monooleate/PEG 40 Castor oil	118.0 ± 37.0
(4) Sorbitan monooleate/PEG 54 Castor oil	187.0 ± 34.0
(5) PEG 15 Castor oil/PEG 30 Castor oil	63.0 ± 10.0
(6) PEG 15 Castor oil/PEG 36 Castor oil	72.0 ± 16.0
(7) PEG 15 Castor oil/PEG 40 Castor oil	64.0 ± 16.0
(8) PEG 15 Castor oil/PEG 54 Castor oil	63.0 ± 16.0

(*) SD = standard deviation.

**Table 3:** Nanoemulsion stability according different temperature values.

Temperature ( ±1 °C)									
Formulations	40	45	50	55	60	65	70	75	80
1 (T_MOS_/T_30OE_)	n	n	n	sl	sl	sl	sl	sl	sl
2 (T_MOS_/T_36OE_)	n	n	n	n	sl	sl	sl	sl	sl
3 (T_MOS_/T_40OE_)	n	n	n	n	n	n	sl	sl	sl
4 (T_MOS_/T_54OE_)	n	n	n	n	n	n	n	n	n
5 (T_15OE_/T_30OE_)	n	n	n	sl	sl	sl	sl	sl	sl
6 (T_15OE_/T_36OE_)	n	n	n	sl	sl	sl	sl	sl	sl
7 (T_15OE_/T_40OE_)	n	n	n	sl	sl	sl	sl	sl	sl
8 (T_15OE_/T_45OE_)	n	n	n	sl	sl	sl	sl	sl	sl

n= normal; sl = slight modification; MOS = sorbitan monooleate; OE = ethylene oxide.

## References

[1] Tadros T., Izquierdo P., Esquena J., Solans C. (2004). Formation and stability of nano-emulsions. Adv. Colloid Interface Sci..

[2] Maruno M., Rocha-Filho P.A. (2010). O/W Nano-emulsion after 15 Years of Preparation: A Suitable Vehicle for Pharmaceutical and Cosmetic Applications. J. Dispers. Sci. Technol..

[3] Stone L. (2000). Medilan: A hypoallergenic lanolin for emollient therapy. Br. J. Nurs..

[4] Porras M., Solans C., González C., Martínez A., Guinart A., Gutiérrez J.M. (2004). Studies of formation of W/O nano-emulsions. Colloids Surf. A Physicochem. Eng. Asp..

[5] Martins E.F., Pereira L.M., Lima T.M., Aguiar G.R., Chen S.C., Folador A., Curi T.C.P., Curi R. (2005). Influência da lanolina na cicatrização. Saúde Rev..

[6] Morais J.M., Santos O.D.H., Delicato T., Gonçalves R.A., Rocha-Filho P.A. (2006). Physicochemical characterization of canola oil/water nano-emulsions obtained by determination of required HLB number and emulsion phase inversion methods. J. Dispers. Sci. Technol..

[7] Pey C.M., Maestro A., Solé I., Gonzalez C., Solans C. (2006). Optimization of nano-emulsions prepared by low-energy emulsification methods at constant temperature using a factorial desing study. Colloids Surf. A Physicochem. Eng. Asp..

[8] Peng L.C., Liu C.H., Kwan C.C., Huang K.F. (2010). Optimization of water-in-oil nano-emulsions by mixed surfactants. Colloids Surf. A Physicochem. Eng. Asp..

[9] Rocha-Filho P.A., Maruno M., Oliveira B., Bernardi D.S., Gumiero V.C., Pereira T.A. (2014). Nanoemulsions as a Vehicle for Drugs and Cosmetics. Nanosci. Technol..

[10] Pereira T.A. (2011). Obtenção e caracterização de nanoemulsões O/A à base de óleo de framboesa, maracujá e pêssego: Avaliação de propriedades cosméticas da formulação. Tese de Mestrado—Faculdade de Ciências Farmacêuticas de Ribeirão Preto.

[11] Fernandez P., Andre V., Rieger J., Kuhnle A. (2004). Nano-emulsions formation by emulsion phase inversion. Colloids Surf. A Physicochem. Eng. Asp..

[12] Hessien M., Singh N., Kim C., Prouzet E. (2016). Stability and Tunability of O/W nano-emulsions prepared by phase inversion composition. Langmuir.

[13] Solans C., Izquierdo P., Nolla J., Azemar N., Celma M.J.G. (2005). Nano-emulsions. Curr. Opin. Colloid Interface Sci..

[14] Maestro A., Solé I., González C., Solans C., Gutiérrez J.M. (2008). Influence of the phase behavior on the properties of ionic nano-emulsions prepared by the phase inversion composition method. J. Colloid Interface Sci..

[15] Liu W., Sun D., Li C., Liu Q., Xu J. (2006). Formation and stability of paraffin oil-in-water nano-emulsions prepared by emulsion inversion point method. J. Colloid Interface Sci..

[16] Sajjadi S. (2007). Formation of fine emulsions by emulsification at high viscosity or low interfacial tension: A comparative study. Colloids Surf. A Physicochem. Eng. Asp..

[17] Spinelli L.S., Mansur C.R.E., González G., Lucas E.F. (2010). Evaluation of process conditions and characterization of particle size stability of oil-in-water nano-emulsions. Colloid J..

[18] Yang H.J., Cho W.G., Park S.N. (2009). Stability of oil-in-water nano-emulsions prepared using the phase inversion composition method. J. Ind. Eng. Chem..

[19] Chiesa M., Garg J., Kang Y.T., Chen G. (2008). Thermal conductivity and viscosity of water-in-oil nano-emulsions. Colloids Surf. A Physicochem. Eng. Asp..

[20] Morais G.C., Szantos O.D.H., Oliveira W.P., Rocha-Filho P.A. (2008). Attainment of O/W emulsions containing liquid crystal fromannatto oil (*Bixa orellana*), coffee oil, and tea tree oil (*Melaleuca alternifolia*) as oily phase using HLB system and ternary phase diagram. J. Dispers. Sci. Technol..

[21] Teo B.S.X., Basri M., Zakaria M.R.S., Salleh A.B., Rahman R.N.Z.R.A., Rahman M.B.A. (2010). A potential tocopherol acetate loaded palm oil esters-in-water nano-emulsions for nanocosmeceuticals. J. Nanobiotechnol..

[22] Aulton M.E. (2005). Delineamento de Formas Farmacêuticas.

[23] Weiss J., Coupland J.N., Mcclements D.J. (1996). Solubilization of hydrocarbon emulsion droplets suspended in non- ionic surfactant micelle solutions. J. Phys. Chem..

[24] Masmoudi H., le Dréau Y., Piccerelle P., Kister J. (2005). The evaluation of cosmetic and pharmaceuticalemulsions aging process using classical techniques and a new method: FTIR. Int. J. Pharm..

[25] Ramalho V.C., Jorge N. (2006). Antioxidantes usados em óleos, gorduras e alimentos gordurosos. Quím. Nova.

[26] Ee S.L., Duan X., Liew J., Nguyen D. (2008). Droplet size and stability of nano-emulsion produced by the temperature phase inversion method. Chem. Eng. J..

[27] Wissing S.A., Muller R.H. (2003). The influence of solid lipid nanoparticles on skin hydration and viscoelasticity—*In vivo* study. Eur. J. Pharm. Biopharm..

[28] Pianovski A.R., Vilela A.F.G., Silva A.A.S., Lima C.G., Silva K.K., Carvalho V.F.M., Musis C.R., Machado S.R.P., Ferrari M. (2008). Desenvolvimento e avaliação da estabilidade de emulsões múltiplas O/A/O com óleo de pequi (*Caryocar brasiliense*). Rev. Bras. Farm..

[29] Bernardi D.S., Pereira T.A., Maciel N.R., Bortoloto J., Viera G.S., Oliveira G.C., Rocha-Filho P.A. (2011). Formation and stability of oil-in-water nanoemulsions containing rice bran oil: *In vitro* and *in vivo* assessments. J. Nanobiotechnol..

